# The Use of Virtual Reality to Facilitate Mindfulness Skills Training in Dialectical Behavioral Therapy for Borderline Personality Disorder: A Case Study

**DOI:** 10.3389/fpsyg.2016.01573

**Published:** 2016-11-02

**Authors:** Maria V. Nararro-Haro, Hunter G. Hoffman, Azucena Garcia-Palacios, Mariana Sampaio, Wadee Alhalabi, Karyn Hall, Marsha Linehan

**Affiliations:** ^1^Hospital General de CatalunyaBarcelona, Spain; ^2^Virtual Reality Research Center at the Human Photonics Lab, Mechanical Engineering, University of Washington SeattleSeattle, WA, USA; ^3^Virtual Reality Research Center, Computer Science Department, Effat UniversityJeddah, Saudi Arabia; ^4^Psychology, Universitat Jaume ICastellon, Spain; ^5^Ciber Bisiopatologia Obesidad y NutricionMadrid, Spain; ^6^Faculty of Computing and Information Technology, King Abdulaziz UniversityJeddah, Saudi Arabia; ^7^The DBT Center of HoustonHouston, TX, USA; ^8^Behavioral Research & Therapy Clinics, University of WashingtonSeattle, WA, USA

**Keywords:** virtual reality, borderline personality, mindfulness, dialectical behavioral therapy, emotion regulation

## Abstract

Borderline personality disorder (BPD) is a severe mental disorder characterized by a dysfunctional pattern of affective instability, impulsivity, and disturbed interpersonal relationships. Dialectical Behavior Therapy (DBT®) is the most effective treatment for Borderline Personality Disorder, but demand for DBT® far exceeds existing clinical resources. Most patients with BPD never receive DBT®. Incorporating computer technology into the DBT® could help increase dissemination. Immersive Virtual Reality technology (VR) is becoming widely available to mainstream consumers. This case study explored the feasibility/clinical potential of using immersive virtual reality technology to enhance DBT® mindfulness skills training of a 32 year old female diagnosed with BPD. Prior to using VR, the patient experienced difficulty practicing DBT® mindfulness due to her emotional reactivity, and difficulty concentrating. To help the patient focus her attention, and to facilitate DBT® mindfulness skills learning, the patient looked into virtual reality goggles, and had the illusion of slowly “floating down” a 3D computer-generated river while listening to DBT® mindfulness training audios. Urges to commit suicide, urges to self harm, urges to quit therapy, urges to use substances, and negative emotions were all reduced after each VR mindfulness session and VR mindfulness was well accepted/liked by the patient. Although case studies are scientifically inconclusive by nature, results from this feasibility study were encouraging. Future controlled studies are needed to quantify whether VR-enhanced mindfulness training has long term benefits e.g., increasing patient acceptance and/or improving therapeutic outcome. Computerizing some of the DBT® skills treatment modules would reduce cost and increase dissemination.

## Introduction

Borderline personality disorder (BPD) is a severe mental disorder characterized by a dysfunctional pattern of affective instability, impulsivity, and disturbed interpersonal relationships (American Psychiatric Association, 2013). People with BPD are at increased risk of self-harm, suicidal behaviors, completed suicides, and often have co-occurring disorders, such as depression, anxiety disorders, substance abuse, and eating disorders (Lieb et al., 2004). BPD has been conceptualized as a disorder of the emotion regulation system (Linehan, 1993; Crowell et al., 2009). Even when they try to modulate their own emotions, people with BPD have high negative emotions as their baseline emotional state, they are unusually sensitive, respond intensely, and are slow to calm down (Linehan, 2015).

### Dialectical behavioral therapy

DBT® teaches patients skills to help them control intense emotions, to reduce self-destructive behaviors, and improve relationships. Dialectical Behavioral Therapy (DBT®) is the most effective treatment for individuals with BPD (Stoffers et al., 2012). DBT® includes four intervention modes: individual psychotherapy, phone coaching, a therapist consultation team, and skills training.

### Dialectical behavioral therapy skills training

DBT® skills training covers four main categories: mindfulness, emotion regulation, interpersonal effectiveness, and distress tolerance (Linehan, 1993, 2015). Patients are encouraged to use mindfulness in daily activities. DBT®, including DBT® skills training, aims to reduce patients' difficulties in regulating emotions: impulse control, interpersonal relationships, and self image (Linehan, 2015). As described by Linehan (2015), DBT® Mindfulness skills training is an especially important core component of DBT® that is taught first and helps set the stage for learning other DBT skills. DBT Mindfulness Skills training aims to help individuals change maladaptive patterns of behaviors, emotions, thinking, and relationships with other people that cause problems in their day to day lives. Suicide is one way patients have tried to deal with these severe problems. DBT® skills are aimed to reduce these dysfunctional patterns and help steer patients toward “a life worth living.” The current study focuses on two mindfulness skills, “Observing” skills, and “Wise Mind” skills. “Observing skills” involve learning to pay attention, on purpose, to the present moment: learning to control/focus their attention. In “observing sounds,” patients practice noticing sounds, and practice bringing their attention back to the sounds when their attention wanders. In “observing visuals,” the patient is invited to attend to what they see, without letting their attention get fixed on any one object. The Observing skills exercises include “coming back to your senses,” “focusing the mind” and “opening the mind.” The second mindfulness skill trained in the current study, wise mind, is the “synthesis or integration of opposites: emotion mind and reasonable mind” (Linehan, 2015, p. 169). Mindfulness skills are the vehicles for balancing “emotion mind” and “reasonable mind” to achieve “wise mind” (Linehan, 2015). In previous research, DBT® skills training has been shown to contribute to DBT®'s efficacy for BPD, e.g., significantly higher rate of completion of therapy for patients who received standard DBT® with skills training compared to DBT® without skills training (Linehan et al., 2015). DBT® Mindfulness skills training has also been associated with health benefits such as increased attention and reduced impulsivity (Soler et al., 2012).

Despite its benefits, learning to practice mindfulness is not easy for individuals with BPD. Most patients with BPD have had numerous adverse experiences such as childhood sexual and physical abuse, abandoned relationships, and broken families. BPD patients typically avoid and suppress internal experiences, emotions, and thoughts. Suppression and avoidance contribute to BPD. Mindfulness promotes a patient's awareness of experiences in the present moment without judging and with acceptance. Mindfulness helps patients learn to relate to their unpleasant memories and experiences without judging them. Patients with BPD often have attention deficits that make it harder for them to focus their attention (Soler et al., 2012; McClure et al., 2016). DBT® Mindfulness skills modules help train patients how to better control their attention by giving patients' practice directing their attention to specific content, starting with simple external observations, and gradually progressing to becoming more aware and more in control of their own inner thoughts and emotions. A technology that could help patients focus their attention during mindfulness skills training, that could also potentially increase cooperation on completing DBT® mindfulness skills home works, would be valuable.

Although DBT® is becoming increasingly available, demand for DBT® greatly exceeds existing clinical resources. BPD is a severe disorder, and DBT® treatment is effective at altering the patient's pathological thoughts and behaviors, but the treatment is very intensive. Most patients with BPD never receive DBT®. Although one-on-one therapy is crucial, research is needed on how to provide therapists with effective therapy delivery technologies that can reduce therapist burnout, reduce costs (Castelnuovo et al., 2016) and increase the number of patients who can benefit from DBT®.

Incorporating emerging computer technologies such as *immersive virtual reality* into the DBT® could help increase dissemination, reduce suffering, and reduce suicides via increased access to DBT®. Immersive virtual reality is designed to give participants the illusion of going inside the 3D computer generated virtual world as if it is a place they are visiting (e.g., giving participants the illusion of descending down a 3D computer generated river in a virtual canoe).

### Immersive virtual reality (VR)

Immersive VR is proving useful as a new tool clinical psychology therapists can use to enhance the effectiveness of cognitive-behavior therapy to treat many psychological disorders. Cognitive Behavioral Therapy using VR exposure therapy has been shown to be efficacious in the treatment of anxiety disorders (Opris et al., 2012), like spider phobia (Garcia-Palacios et al., 2002; Hoffman et al., 2003), fear of flying (Rothbaum et al., 1996; Krijn et al., 2004), social phobia (Kampmann et al., 2016), small animal phobia (Botella et al., 2016), and claustrophobia (Botella et al., 1999). Cognitive Behavioral Therapy involving VR exposure is effective for treating more severe psychological disorders such as Post-Traumatic Stress Disorder (Rothbaum et al., 2001; Difede and Hoffman, 2002; Freedman et al., 2010; Rizzo et al., 2010; Difede et al., 2014). VR has also been used to treat eating disorders (Manzoni et al., 2016; Wiederhold et al., 2016) and may help treat delusions (Freeman et al., 2016), self-criticism (Falconer et al., 2014); and patients with chronic pain (Botella et al., 2013; Garcia-Palacios et al., 2015). To our knowledge; no studies have tested VR as an intervention to facilitate mindfulness skills training for DBT®, nor are there any studies using VR to treat BPD, an unusually severe mental disorder.

Virtual Reality is so attention grabbing that it is being used to distract burn patients from their pain during burn wound care, as a non-pharmacologic analgesic (Hoffman, 2004, 2011) and can distract claustrophobics during mock fMRI brain scans (Garcia-Palacios et al., 2007a). Immersive VR blocks the patients' view of the real world, and may help patients focus their attention on the mindfulness skills exercise in VR. VR could also potentially contribute to treatment efficacy via generalization of learned skills to real life practice (Swan et al., in press) and may increase patient acceptance of therapy (Garcia-Palacios et al., 2007b; Botella et al., 2015).

The main objective of the present study is to explore the feasibility and the clinical potential of using VR to facilitate DBT® mindfulness skills learning in a BPD patient. The hypotheses of the current study are: (1) The participant will accept VR for learning the mindfulness skills of DBT®; (2) DBT® mindfulness skills training in virtual reality will decrease BPD-related urges, (e.g., will decrease urges to commit suicide), and will decrease negative emotions (e.g., anger) after each VR mindfulness session; (3) VR+DBT® mindfulness skills training will increase observing, awareness, non-judgmental skills, and positive emotions after each VR mindfulness session.

## Materials and methods

This study was approved by the University of Washington IRB, and the participant signed a consent form. The participant was not compensated for their participation in this study.

### Participant

The participant was a 32-year-old woman diagnosed with BPD and Substance Use Disorder receiving standard DBT®. At the time of the screening, the participant was unemployed; her social support was low and she reported being very dependent on her boyfriend, who also had a Substance Use Disorder diagnosis. The participant reported a 4-year history of poly-substance dependence and multiple hospitalizations due to drug overdose. She had a suicide attempt and two severe non-suicidal self-injuries during the previous 6 months. Patients treated at the Behavior Research and Therapy Clinics (BRTC) often have very severe symptoms, so symptom severity was not the reason the patient was selected for the present case study. The patient was selected because she had trouble practicing mindfulness. Before entering the current Virtual Reality (VR) study, the participant showed difficulties practicing mindfulness via standard DBT® mindfulness training delivered both in individual and group therapy. She showed emotional reactivity, low ability to concentrate, and had trouble doing her mindfulness homework exercises. Thus, the DBT® team thought this patient would be a good candidate to receive the VR+DBT® mindfulness skills training.

### Therapists

The participant was treated by the first author of this paper. The therapist has a Ph.D. in clinical psychology and was working as a postdoctoral fellow at the Behavioral Research and Therapy Clinics, Department of Clinical Psychology, University of Washington, USA http://blogs.uw.edu/brtc/at the time this study was conducted. She treated the participant as part of the BRTC clinical DBT® team during the treatment and attended a weekly DBT® consultation team meeting supervised by Dr. Linehan. The treating therapist was supervised during the treatment of the participant by a senior DBT® trained therapist.

### Design

This was a within-subject design case study with key measures administered before and after each VR mindfulness training session.

### Study procedures

After 1 month of standard DBT® (with no VR), VR+DBT® mindfulness skills learning (Observing skills and Wise Mind skills) was integrated into four individual therapy one-on-one sessions. The standard DBT® program consists of four modes of treatment (individual therapy, group skills training, phone coaching and consultation team meetings) that are delivered once a week. During the first month of standard DBT®, the patients receive a pre-treatment phase in which they are committed to the therapy and the goals of the treatment are set up. Thus, this client started VR at the approximate time she finished the pre-treatment phase.

The measures were administered before and after each VR intervention. During the VR+DBT® skills learning interventions, the participant looked into a pair of wide field of view VR goggles (80 degrees field of view diagonal, per eye), and had the illusion of floating down a 3-D computer-generated river (created and owned by bigenvironments.com, see also vrpain.com) while listening to one of three DBT® mindfulness training audio tracks. (All audio tracks and verbal DBT® treatments used in this study are copyrighted by Marsha Linehan). See below a brief description of each audio track as well as the VR system.

Track 1: Observing Sound (Linehan, 2002; 8.5 min total).

While the patient is in VR, the audio explains briefly for about 3.5 min that most people either confuse mindfulness with relaxation or expect to feel better after mindfulness practice. She asserts that the goal of mindfulness is not necessarily to feel better, but to just notice. The 5-min practice consists of noticing sounds, and repeatedly bringing attention back to sounds every time the mind wanders off.

Track 2: Observing visuals: (Linehan, 2015; 10 min total).

This is a new audio created for the current study to synchronize with images the patient sees in the VR goggles while they are listening to the audio. At the beginning, the audio spends about 1 min explaining the instructions of the exercise. The remaining 9 min are a combination of guided and silent practice. In the guided section, the person is invited to attend to what he/she sees in the virtual world (e.g., the trees, the sky, the intensity of colors). The goal is for the client to just observe, to notice, not to let their attention get fixed upon anything, just watch the things along the way, learning to bring attention back, if it becomes distracted.

Track 3: Wise Mind; (Linehan, 2002; 8 min long).

The patient listens to this audio while in VR floating down the river. This recording starts with a 1-min brief explanation of the concept of wise mind and the instructions about the practice. This particular exercise is called “Stone flake on the lake” (Linehan, 2015). The exercise consists of a 7-min practice where the person is instructed to imagine that he/she is a stone, who is floating down an imaginary lake toward the bottom of the lake, which represents the inner wise mind.

#### Virtual reality system

Toward the goal of creating an immersive VR system (Slater and Wilbur, 1997), the current study used a High Tech VR system designed to (1) shut out physical reality, using VR goggles and headphones that excluded sights and sounds from the real world, (2) providing converging evidence to multiple senses (sights and sounds), (3) providing a surrounding panoramic view rather than a limited narrow field of view (4) vivid high resolution, (5) permitting the participant to interact with the virtual world using a computer mouse to look around in the virtual world.

**Figure 1:**
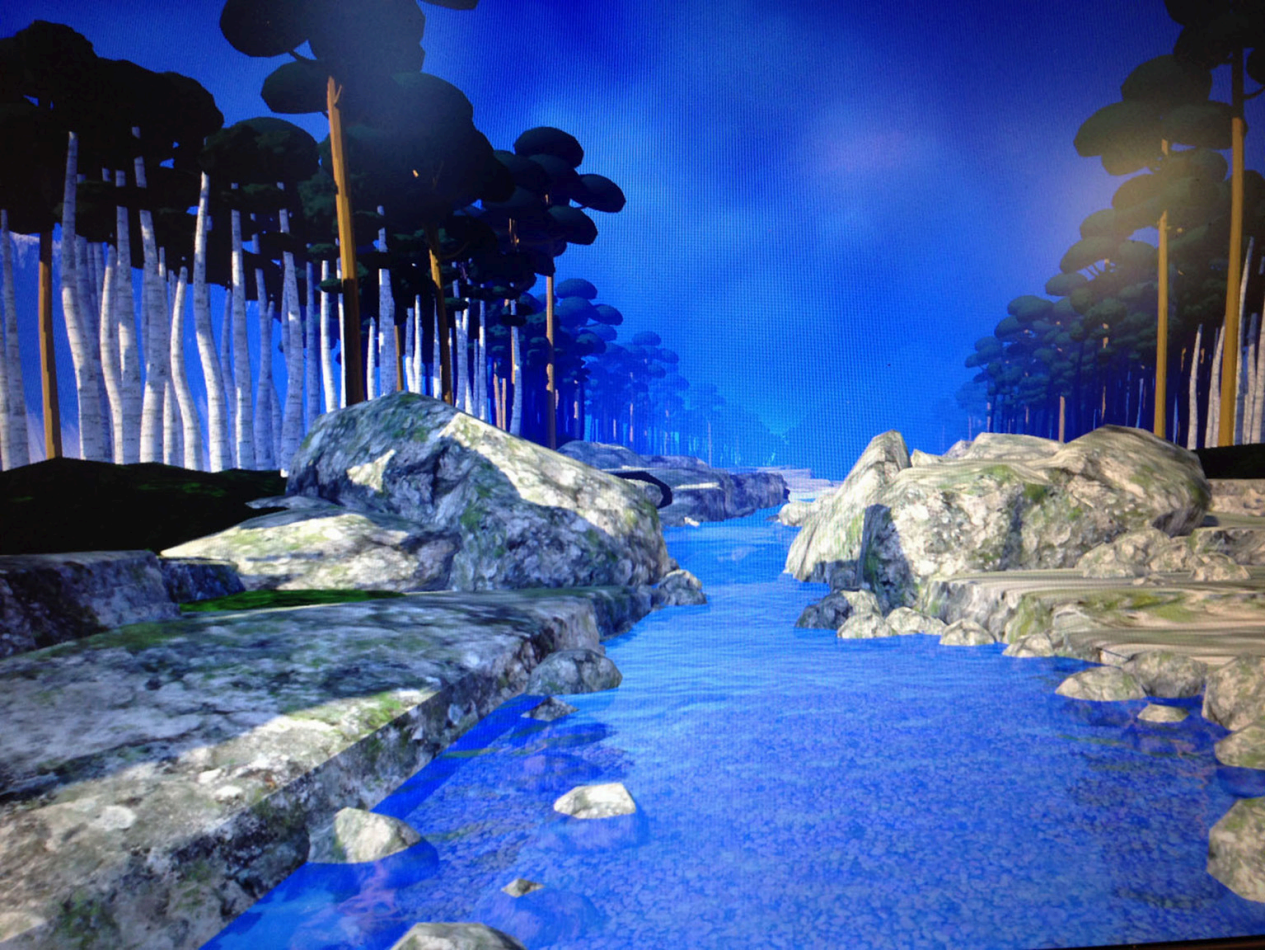
**A screenshot of the Virtual reality world the patient watched through Oculus Rift DK2 VR goggles while listening to Dr. Linehan's DBT^®^ Mindfulness Skills™ audios (http://behavioraltech.org)**. Image by www.bigenvironments.com, copyright Hunter Hoffman, www.vrpain.com.

The participant sat in a chair and looked into a pair of Kaiser Electro-Optics VR goggles with 80 degrees diagonal field of view per eye and 1280 × 1024 resolution per eye. The VR world (see Figure 1) was designed to give the patient the illusion of going inside the 3D computer generated world, where they floated slowly down a computer generated river in VR, with trees, boulders, and mountains (VR World/visuals created and copyrighted by BigEnvironments.com using Unity3D software, see also vrpain.com). The patient listened to Observing and Wise Mind audios while in virtual reality, and one audio (“observing visuals”) was customized to synchronize with what the patient was seeing in VR. The patient received “observing visuals” for session 1, “wise mind” for session 2, “observing sound” for session 3, and received “observing visuals” again for session 4.

## Assessments

To measure BPD-related urges and primary emotions, a form was adapted by the authors using the DBT® diary card (Linehan, 1993), a measure that tracks mood, urges and dysfunctional behaviors. Before and after each VR mindfulness session, the patient briefly rated on a scale from zero to five their current urges to self-injure, their urges to commit suicide, and their urges to use substances, and she rated the intensity (range = 0–100) of several primary emotions (sadness, fear, anger, guilt, shame, disgust, and joy). To measure the patient's acceptance of using VR technology to learn mindfulness skills, the patient gave comments about how she was doing and about her experience and opinions about VR before and after VR DBT Mindfulness Skills training. The KIMS-Short (Höfling et al., 2011) is a 20 item self-report questionnaire designed to measure mindfulness skills. This questionnaire includes the four subscales of the original version: Observing, Describing, Act with Awareness, and Accept without Judgment, and adds one more subscale by separating the original Observing dimension in two: “Observing in” and “Observing out.” In the present study, the KIMS-Short was used as an exploratory measure to measure current state of mindfulness during the VR session.

## Results

Prior to being enrolled in the current study and trying VR, the participant showed difficulties practicing mindfulness (i.e., emotional reactivity, low ability to concentrate). According to the patients written comments (e.g., Supplementary Materials, Table A), and her comments during sessions, VR mindfulness DBT® skills training was well accepted by the patient. She was willing to try the technique, and had a good experience. She said it helped her focus, helped her practice mindfulness and helped her generalize the practice to her natural context outside of therapy, e.g., she started to practice mindfulness at a lake near her house.

Results of patient's BPD-related urges are shown in Table 1. As shown in Table 1 below, Urges to commit suicide, urges to self-harm, urges to quit therapy, and urges to use substances, were reduced after VR+DBT® mindfulness when they were present before the VR mindfulness session, for each of the four VR DBT® Mindfulness sessions (one VR session per week for 4 weeks).

**Table 1:** **Urges (0–5) before and after each VR DBT^®^ Mindfulness session**.

**Condition**	**Period**	**Urge to commit suicide**	**Urge to self-harm**	**Urge to quit therapy**	**Urge to use substances**
Session 1: Observing visuals	Pre-VR	0	0	0	3
	Post-VR	0	0	0	2
Session 2: Wise mind	Pre-VR	3	2	3	4
	Post-VR	0	0	0	3
Session 3: Observing sounds	Pre-VR	0	0	1	3
	Post-VR	0	0	0	2
Session 4: Observing visuals	Pre-VR	0	0	1	4
	Post-VR	0	0	0	3

**Figure 2:**
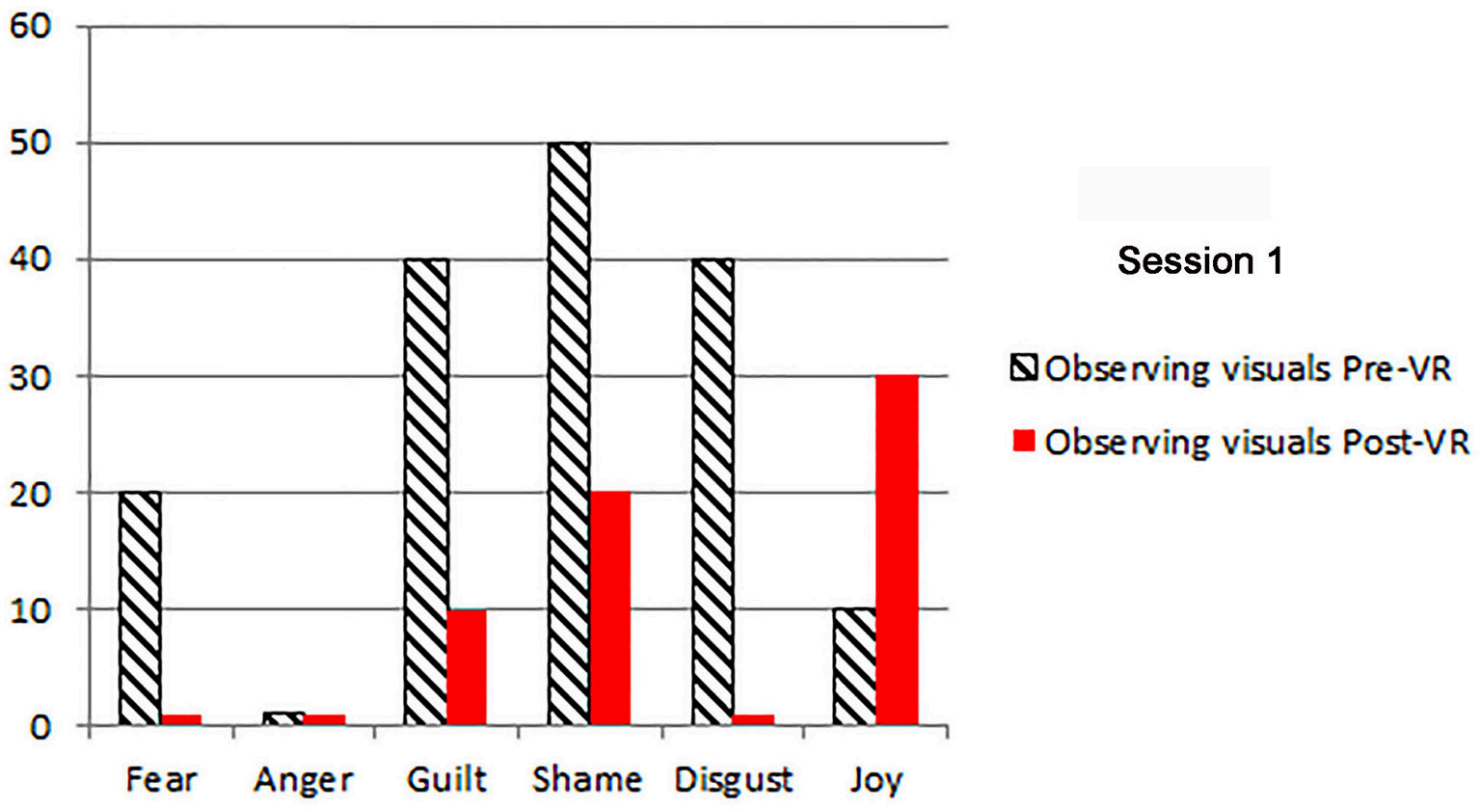
**The patient's primary emotions “right now” (0 − 100), before and after VR+DBT® Mindfulness session 1 (Observing visuals)**.

**Figure 3:**
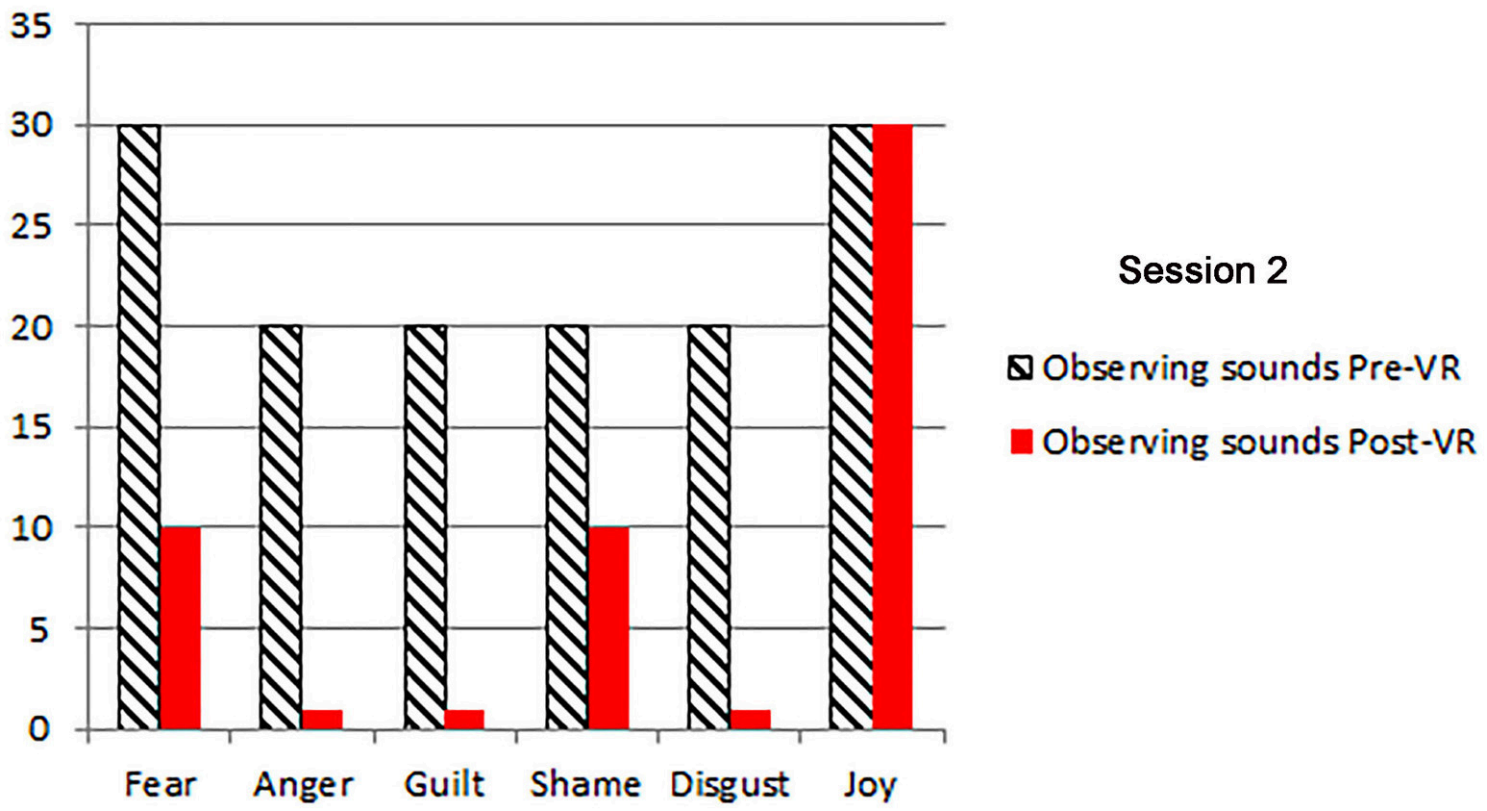
**The patient's primary emotions “right now” (0 − 100), before and after VR+DBT® Mindfulness session 2 (Observing sounds)**.

**Figure 4:**
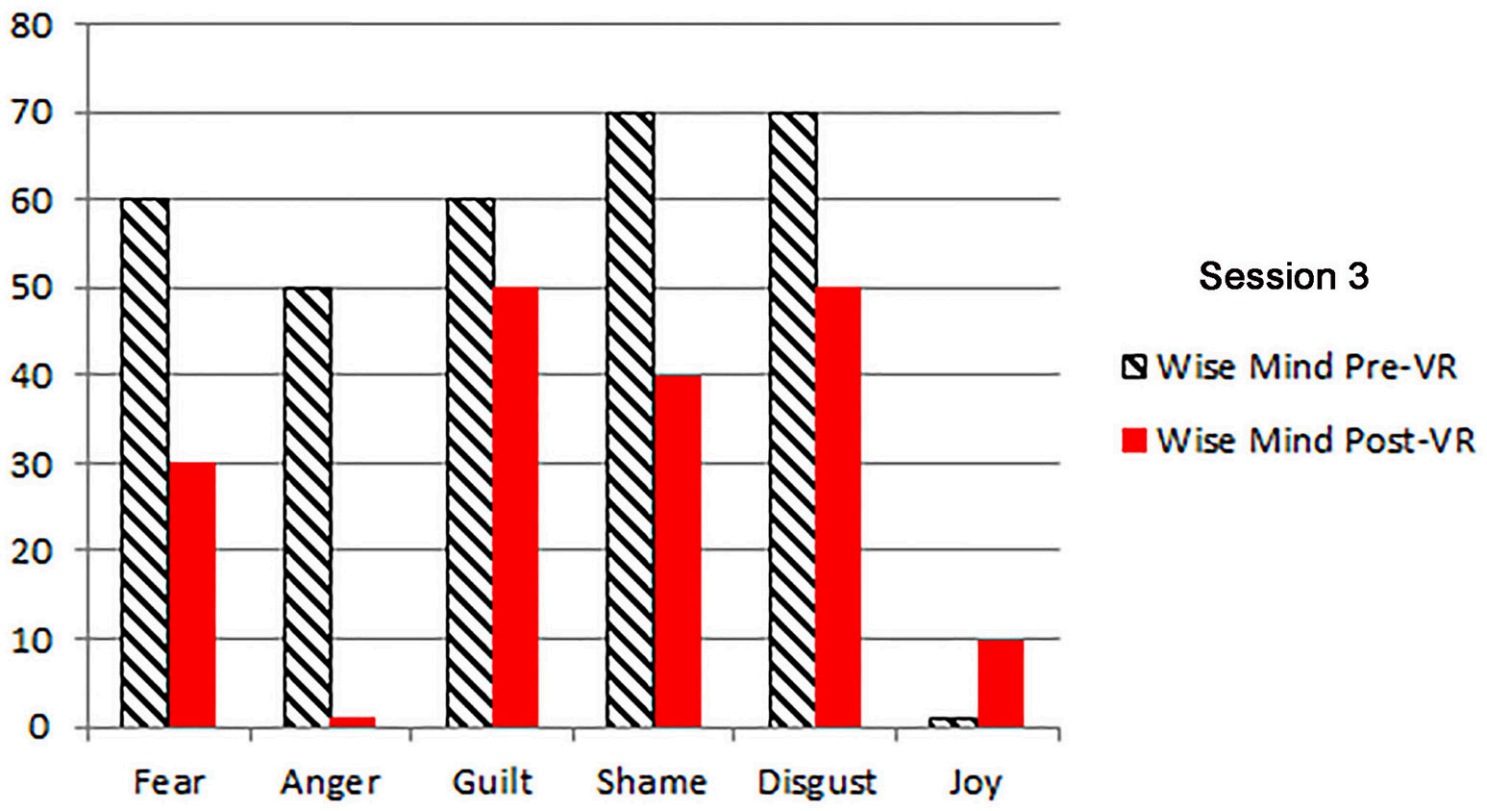
**The patient's primary emotions “right now” (0 − 100), before and after VR+DBT® Mindfulness session 3 (Wise mind)**.

**Figure 5:**
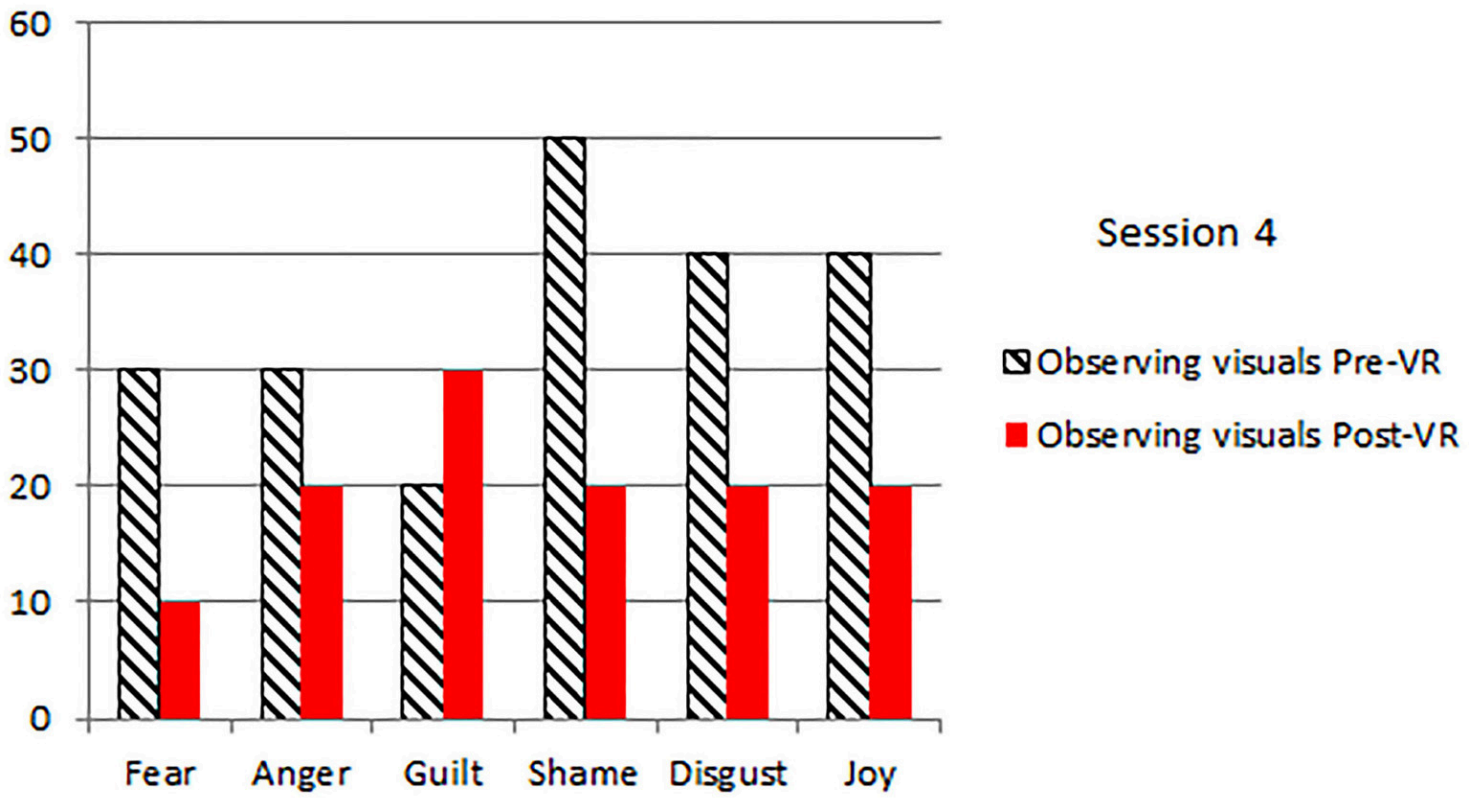
**The patient's primary emotions “right now” (0 − 100), before and after VR+DBT® Mindfulness session 4 (Observing visuals)**.

The patients primary emotions before and after each VR session, measured using an adaptation of the *DBT*^®^ *diary card*, (Linehan, 1993) are shown in Figures 2–5. As predicted, the three different VR mindfulness exercises were effective for reducing negative emotion (fear, anger, guilt, shame, and disgust) after VR mindfulness training.

As shown in Supplementary Materials, Table B, mindfulness skills, and total mindfulness scores were measured using an exploratory adaptation of the KIMS-Short version (by asking patients to rate their current state of mindfulness). Results were in the predicted direction (increasing the mindfulness scores) for Wise Mind and Observing sounds VR sessions, but were slightly in the opposite of the predicted direction (a slight decrease) after the Observing Visuals exercise.

## Discussion

The current case study shows encouraging preliminary evidence that the first patient with BPD to try VR enhanced mindfulness training, accepted the use of VR as part of their treatment and showed improved emotional state after the VR sessions. She was willing to try DBT® virtual reality mindfulness, and had a good experience. She said it helped her focus, helped her practice mindfulness, and helped her generalize the practice of mindfulness to her natural context outside of therapy, e.g., she started to practice mindfulness at home.

Even when they try to modulate their own emotions, people with BPD have high negative emotions as their baseline emotional state, they are unusually sensitive, respond intensely, and are slow to calm down (Linehan, 2015). The VR+DBT® mindfulness practice helped the participant reduce current negative emotions very common in BPD patients: sadness, fear, anger, guilt, shame and disgust. The VR + DBT® mindfulness skills training also reduced the patient's urges to commit suicide, urges to self-harm, urges to quit therapy, and urges to use substances were also decreased after each VR + DBT® Mindfulness Skills training session.

As for mindfulness skills measured with the exploratory KIMS-Short scale, observing sounds and wise mind VR exercises produced an improvement in the total scores of mindfulness from pre-VR session to post-VR session, but scores were slightly in the opposite of the predicted direction (a slight decrease) after the observing visuals exercise.

Traditional DBT® skills training (with no VR) has been shown to contribute to DBT®'s efficacy for BPD (Linehan et al., 2015). We predict that, with further development, using this and other VR systems (Hoffman et al., 2003, 2006, 2014; Wender et al., 2009; Wendrich et al., 2016), immersive virtual reality and augmented reality can enhance DBT® skills training, and could further increase the effectiveness of DBT® for BPD. DBT®-VR systems that patients can take home and use in their own homes, potentially via networked multi-participant VR (Wendrich et al., 2016), could be valuable for increasing compliance with homework assignments.

Borderline personality disorder is a very severe mental disorder, challenging to treat. “Although DBT® is clearly efficacious and increasingly available in practice settings, demand for DBT® far exceeds existing resources” (Linehan et al., 2015, p 476). Most people with BPD never receive DBT®. Increasing dissemination of DBT® is a high priority. Virtual Reality may be an especially good match for enhancing DBT® mindfulness skills learning because VR helps give patients the illusion of “being there” in the computer generated world (being in a place), and “being in the present moment” (being in a time) is the essence of mindfulness. VR is in the process of becoming commercially available and widely used by mainstream consumers (e.g., Hoffman et al., 2014). VR has the potential to make DBT® skills training more widely available to the public. Computerizing some of the DBT® treatment modules could reduce treatment costs and increase dissemination.

Although this study is promising, it was a case study, and lack of follow up measures is an important limitation. Although case studies are by nature scientifically inconclusive (Campbell and Stanley, 1963), it is an important initial step in the exploration of using VR computer technology to potentiate DBT® skills training. Another possible limitation may be the factor that the patient was receiving a broader program, the standard DBT®, and the results may be influenced by other treatment variables different than the VR mindfulness that she was learning (e.g., skills learned in group or other factors such as therapeutic alliance), however, the within-subjects design should help isolate the current findings to the influence of the VR DBT® mindfulness sessions.

In conclusion, these results show for the first time the feasibility of using immersive virtual reality to facilitate mindfulness practice in a case of BPD.

These results encourage further development, and larger, carefully controlled efficacy studies to determine whether VR mindfulness training has long term benefits (e.g., improved clinical outcome), and to determine whether VR can enhance mindfulness skills training for BPD patients, who often have difficulty focusing their attention during attention training exercises.

## Author contributions

All authors listed, have made substantial, direct, and intellectual contribution to the work, and approved it for publication.

## Funding

This research was supported by Effat University, Jeddah Saudi Arabia Research and Consultancy Institute.

### Conflict of interest statement

ML, ABPP is the treatment developer of Dialectical Behavior Therapy (DBT). She receives licensing fees for Behavioral Tech's use of her training materials. The other authors declare that the research was conducted in the absence of any commercial or financial relationships that could be construed as a potential conflict of interest.
