# Supplementary material for: The Use of Virtual Reality to Facilitate Mindfulness Skills Training in Dialectical Behavioral Therapy for Borderline Personality Disorder: A Case Study
Source: Front Psychol. 2016 Nov 2;7:1573. doi: 10.3389/fpsyg.2016.01573 (PMC5089996; doi:10.3389/fpsyg.2016.01573)
Supplement: Supplementary file 1 [file Table1.PDF]

Supplementary Materials Table A.-Comments by the participant about the practice or about herself:

Session 1: Observing visuals	Pre-VR	No comments collected
	Post-VR	No comments collected
Session 2: Wisemind	Pre-VR	It has been a while since I have been to therapy, feeling guilty about having a hard time with consequences and being able to live with how I feel on a daily basis
	Post-VR	Had a lot of emotions coming into therapy that I felt very stuck in
Session 3: Observing sounds	Pre-VR	Long week, very tired
	Post-VR	I liked the new one with the sounds, helped to visualize, be more present
Session 4: Observing visuals	Pre-VR	Long stressful week, feeling lost, however trying to make things happen for myself
	Post-VR	Great exercise to help day to day
