# Supplementary material for: The Use of Virtual Reality to Facilitate Mindfulness Skills Training in Dialectical Behavioral Therapy for Borderline Personality Disorder: A Case Study
Source: Front Psychol. 2016 Nov 2;7:1573. doi: 10.3389/fpsyg.2016.01573 (PMC5089996; doi:10.3389/fpsyg.2016.01573)
Supplement: Supplementary file 2 [file Table2.PDF]

Supplementary Materials Table B.-KIMS before and after VR DBT Mindfulness

Condition		Act With Awareness	Describe	Accept Without Judgment	Observing IN	Observing OUT	Mindfulness total
Session 1: Observing visuals	Pre-VR	1.75	4.00	3.00	3.33	3.33	3.00
	Post-VR	2.50	3.50	2.60	2.67	2.67	2.76
Session 2: Wisemind	Pre-VR	2.75	3.75	2.80	2.33	2.33	2.82
	Post-VR	2.25	4.00	3.00	3.00	3.33	3.06
Session 3: Observing sounds	Pre-VR	2.00	3.25	3.00	3.00	2.33	2.65
	Post-VR	2.25	3.25	3.00	3.00	2.67	2.76
Session 4: Observing visuals	Pre-VR	2.50	3.75	3.40	2.00	2.67	2.88
	Post-VR	2.00	3.25	3.20	2.67	3.00	2.71
